# Expression profiling and functional analysis of circular RNAs *in vitro* model of intermittent hypoxia-induced liver injury

**DOI:** 10.3389/fphys.2022.972407

**Published:** 2022-09-14

**Authors:** Li-Da Chen, Jie-Feng Huang, Xue-Jun Lin, Ya-Ping Huang, Qiao-Zhen Xu, Gong-Ping Chen, Qi-Chang Lin

**Affiliations:** ^1^ Department of Respiratory and Critical Care Medicine, Zhangzhou Affiliated Hospital of Fujian Medical University, Zhangzhou, China; ^2^ Department of Respiratory and Critical Care Medicine, the First Affiliated Hospital of Fujian Medical University, Fuzhou, China; ^3^ Fujian Provincial Sleep-disordered Breathing Clinic Center, Fuzhou, China; ^4^ Laboratory of Respiratory Disease of the Fujian Medical University, Fuzhou, China; ^5^ Department of Laboratory Medicine, Zhangzhou Affiliated Hospital of Fujian Medical University, Zhangzhou, China

**Keywords:** obstructive sleep apnea, intermittent hypoxia, liver injury, circular RNAs, sequence analysis

## Abstract

Intermittent hypoxia (IH) is a prominent feature of obstructive sleep apnea (OSA) which is increasingly recognized as a key risk factor for liver injury. Circular RNAs (circRNAs) has been suggested to act as a regulator of multiple biological processes. However, there is no study evaluating circRNAs alterations and potential role of circRNAs in OSA-related liver injury. The present study aimed to investigate circRNA expression profiles *in vitro* model of IH-induced liver injury, as well as potential functional characterization of the differentially expressed circRNAs (DE circRNAs). BRL-3A cells were exposed to IH or normoxia. Cell apoptosis and cell viability were evaluated using flow cytometry and cell counting kit-8, respectively. The expression profile of circRNAs was depicted by circRNA sequencing. The selected circRNAs were verified by quantitative real-time PCR (qRT-PCR). Kyoto Encyclopedia of Genes and Genomes (KEGG) pathway and Gene Ontology (GO) analyses were employed to predict DE circRNAs functions. The circRNA-miRNA-mRNA regulatory network was constructed. IH treatment caused cell injury in BRL-3A cells. 98 circRNAs were identified as being dysregulated in IH-treated BRL-3A cells. Among them, 58 were up-regulated and 40 were down-regulated. Go and KEGG analyses suggested that the DE circRNAs were predominantly enriched in the biological process such as positive regulation of NF−kappaB transcription factor activity and pathways such as circadian entrainment, Wnt signaling pathway, MAPK signaling pathway, and protein export. 3 up-regulated circRNAs and 3 down-regulated circRNAs with high number of back-splicing sites were chosen for qRT-PCR validation and were consistent with the sequencing data. CircRNA1056 and circRNA805 were predicted to interact with microRNAs that might thereby regulate downstream genes. The study characterized a profile of dysregulated circRNAs in IH-induced BRL-3A cell injury. DE circRNAs may play vital roles in the pathophysiology of IH-induced liver injury. Our findings provide preliminary support for further research in mechanisms and a new theory for the pathogenesis of OSA-related liver injury.

## Introduction

Obstructive sleep apnea (OSA) is a sleep disorder characterized by repetitive reduction or cessation of airflow due to upper airway collapse. These episodes are associated with chronic intermittent hypoxia (CIH) and sleep fragmentation. Increasing evidence indicates a strong relationship between OSA with liver injury, especially nonalcoholic fatty liver disease (NAFLD) (Musso et al., 2012; Mesarwi et al., 2019; Chen et al., 2021). Our previous studies suggested that OSA was one of key risk factors for liver injury and NAFLD in both adult and pediatric patients (Lin et al., 2015; Chen et al., 2020a). However, the underlying mechanisms linking the two disorders remain largely unknown.

Circular RNAs (circRNAs) are a unique class of RNA molecules which are covalently linked to form a closed circular structure (Memczak et al., 2013). They are characterized by high conservation, abundance, stability, and specificity of tissue development (Lasda and Parker, 2014). CircRNAs are divided into four types: intronic circRNAs, exon-intron circRNAs, exonic circRNAs, and intergenic circRNAs. CircRNAs have recently been shown to exert a vital role in many pathophysiological and physiological processes (Li et al., 2018). The biological function of circRNAs includes microRNA sponge activity, interaction with RNA-binding proteins, modulation of alternative splicing or transcription, rolling circle translation, and circRNA-derived pseudogenes (Liu et al., 2017).

CircRNAs are reported to be involved in a variety of human diseases, such as cancer (Wang et al., 2020a), stroke (Wang et al., 2020b), cardiovascular disorders (Wang et al., 2020c; Mester-Tonczar et al., 2020), neurodegenerative diseases (Kumar et al., 2017), renal diseases (Wu et al., 2021), and liver diseases (Ou et al., 2019). There is no study evaluating circRNAs alterations and potential role of circRNAs in OSA-related liver injury. Intermittent hypoxia (IH) is a prominent feature of OSA pathophysiology. In order to establish *in vitro* model of IH-induced liver injury, BRL-3A cells were exposed to IH. We aim to perform high-throughput RNA sequencing to investigate circRNA expression profiles *in vitro* model of IH-induced liver injury and potential functional characterization of the differentially expressed circRNAs (DE circRNAs). A network of circRNA–miRNA–mRNA was further constructed to investigate the possible regulatory mechanism in IH-induced liver injury.

## Materials and methods

### Cell culture and treatment

BRL-3A cell lines (rat liver cells) were obtained from the Shanghai cell Bank, Chinese Academy of Sciences. Cells were grown in Dulbecco’s modified Eagle’s medium (HyClone) with 10% fetal bovine serum (Gibco) and 1% penicillin/streptomycin. Cells were placed in a cell culture chamber containing 5% CO_2_ at 37°C. To induce IH, cells were incubated in a hypoxic cell culture chamber system (MAWORDE, Longfujia Lifesciences Ltd., Beijing, China). The fractional oxygen concentration (FcO2) was reduced to 1% in the first 8 min, stabilized for 52 min. Then FcO2 in the exposure chamber was increased to 20% in the next 16 min, and stabilized for 14 min (Singh et al., 2019). One cycle of IH lasted for 90 min. The IH period was last for 48 h. The cells in normoxic control (NC) group were incubated in incubator with 5% CO2 and 21% O2 for the same period.

### Cell viability

Cell viability was measured using cell counting kit-8 (CCK-8) kit (Biosharp, China) according to manufacturer’s instructions. BRL-3A cells were seeded at a density of 3× 10^3^ cells per well in 96-well plates and exposed to IH or normoxia condition. Each well was added with 10 ul CCK-8 solution and incubated at 37°C for 2 h. The absorbance at 450 nm was measured using a spectrophotometer.

### Evaluation of apoptosis by flow cytometry

Cell apoptosis was conducted using an Annexin V-FITC/PI apoptosis detection kit (Sangon Biotech, Shanghai, China). After IH or normoxia treatment, BRL-3A cells were collected and washed in phosphate buffered saline (PBS) twice and resuspended in 200 µl binding buffer. Then BRL-3A cells were stained with 10 μL propidium iodide (PI) and 5 μL Annexin V-FITC and incubated for 15 min at room temperature in the dark. BRL-3A cells were further detected using Guava EasyCyte flow cytometer (Merck Millipore). The results were analyzed by FlowJo software (version 10.0.7, TreeStar, Inc.).

### RNA library construction and sequencing

Total RNA was isolated and purified using Trizol reagent (Invitrogen, Carlsbad, CA, United States). NanoDrop ND-1000 (NanoDrop, Wilmington, DE, United States) was used for evaluating the RNA amount and purity. The assessment of RNA integrity was performed using the Agilent 2,100 Bioanalyzer (RNA integrity number >7.0). RNAs were treated with Rnase R (Epicentre Inc, Madison, WI, United States) to remove linear RNAs and to enrich circRNAs after ribosomal RNA-depletion. The enriched circRNAs were fragmented into small pieces using divalent cations under high temperature. The cleaved RNA fragments were reverse-transcribed to generate first-stranded cDNA, and the second-stranded DNAs were next synthesized with *E. coli* DNA polymerase I, RNase H and dUTP. After heat-weakened UDG enzyme treatment, the ligated products were amplified with PCR. The average insert size for the final cDNA library was 300 bp (±50 bp). Sequencing was performed on an Illumina HiSeq 4,000 (LC Bio, China) according to the instructions.

### Bioinformatic analyses

Cutadapt was adapted to remove the reads which contained adaptor contamination, low quality bases and undetermined bases initially. FastQC (http://www.bioinformatics.babraham.ac.uk/projects/fastqc/) was used to verify sequence quality subsequently. Bowtie2 and Tophat2 were used to map reads to the genome of species. Remaining reads were mapped to genome. CIRCExplorer and CIRI were applied to denovo assemble the mapped reads to circRNAs; then, back splicing reads were identified in unmapped reads by tophat-fusion. All samples were generated unique circRNAs. DE circRNAs were identified as those with a |log2 (fold-change)| ≥1 and *p* value < 0.05.

### Gene ontology and kyoto encyclopedia of genes and genomes pathway analyses

To analyze the functions of the DE circRNAs, Gene Ontology (GO) and Kyoto Encyclopedia of Genes and Genomes (KEGG) pathway enrichment analyses on the parental genes of the circRNAs were performed. GO analysis was performed to explore the functional roles of parental gene regarding biological processes (BP), cellular components (CC), and molecular functions (MF). KEGG was used to analyze the related pathways of the parental genes of DE circRNAs. GO terms and KEGG pathways with corrected *p* value < 0.05 were considered significantly enriched.

### RNA extraction and real-time PCR

Total RNA was isolated from BRL-3A cells by the Trizol method according to the manufacturer’s protocol. The cDNA was generated with RevertAid First Strand cDNA Synthesis Kit (Invitrogen). Then, qRT-PCR was performed using SYBR Green qPCR Master Mix (Roche, Switzerland) and a quantitative fluorescence PCR system (ABI 7500 thermocycler, Applied Biosystems, United States). GAPDH was used as an internal control. The relative expression of circRNAs was analyzed using the 2-ΔΔCt method. The primer sequences used were summarized in Supplementary Table S1.

### Construction of a circRNA-miRNA-mRNA regulatory network

Interactions of circRNA-miRNA and miRNA-mRNA, which were predicted using TargetScan and miRanda, were combined to establish a circRNA-miRNA-mRNA network. The networks were constructed based on signal values of circRNAs and miRNAs normalized from the raw profiling data. Cytoscape software was used to visualize this network.

### Statistical analysis

Data analysis was performed using GraphPad Prism 5.0 software (GraphPad, San Diego, CA, United States). All values were expressed as mean ± standard deviation (SD). Student’s t-test was adopted to compare the differences between two groups. Statistical significance was determined as *p* < 0.05.

## Results

### IH treatment induced cell injury in BRL-3A cells

**FIGURE 1:**
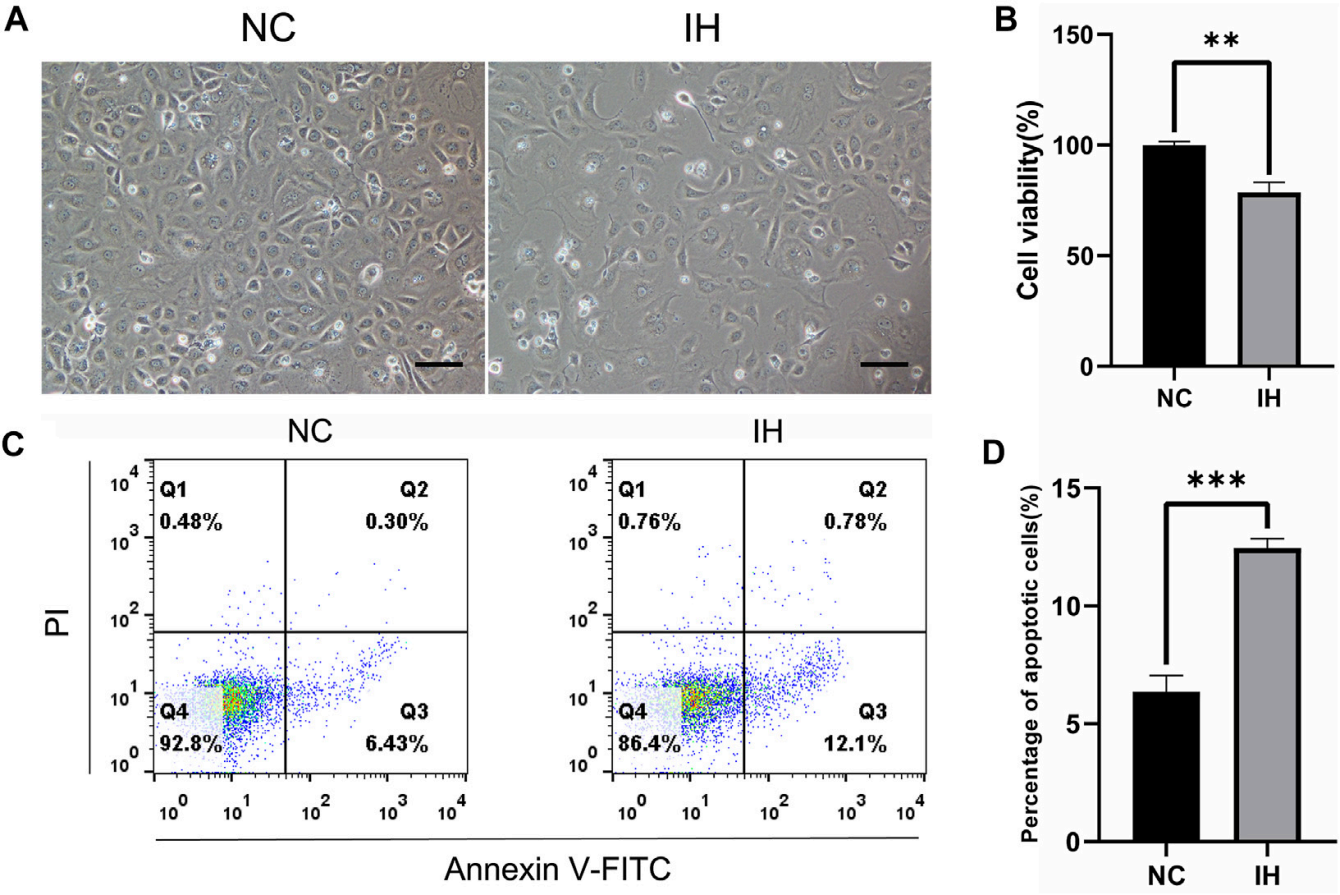
IH treatment induced cell injury in BRL-3A cells. **(A)** Cell morphology images (Original magnification ×100. Bar = 10 μM). **(B)** CCK-8 assay for the determination of cell viability (*n* = 3). **(C)** Cell apoptosis by flow cytometry analysis (*n* = 3). **(D)** Quantitative results of the percentage of cell apoptosis (*n* = 3). **p < 0.01; ***p < 0.001.

To investigate the effect of IH treatment on BRL-3A cells, cell density, cell viability and cell apoptosis rate were compared between normoxic and IH condition. The light microscope picture showed that the cell density decreased obviously in IH group when compared to NC group (Figure 1A). The results of cell viability revealed that there was a significant reduction of cell viability in BRL-3A cells under IH condition (Figure 1B). Furthermore, Flow cytometry assay revealed that IH treatment significantly aggravated cell apoptosis rate (Figures 1C,D). These data suggested that IH treatment led to liver cell injury.

### Expression profiles of circRNAs

**FIGURE 2:**
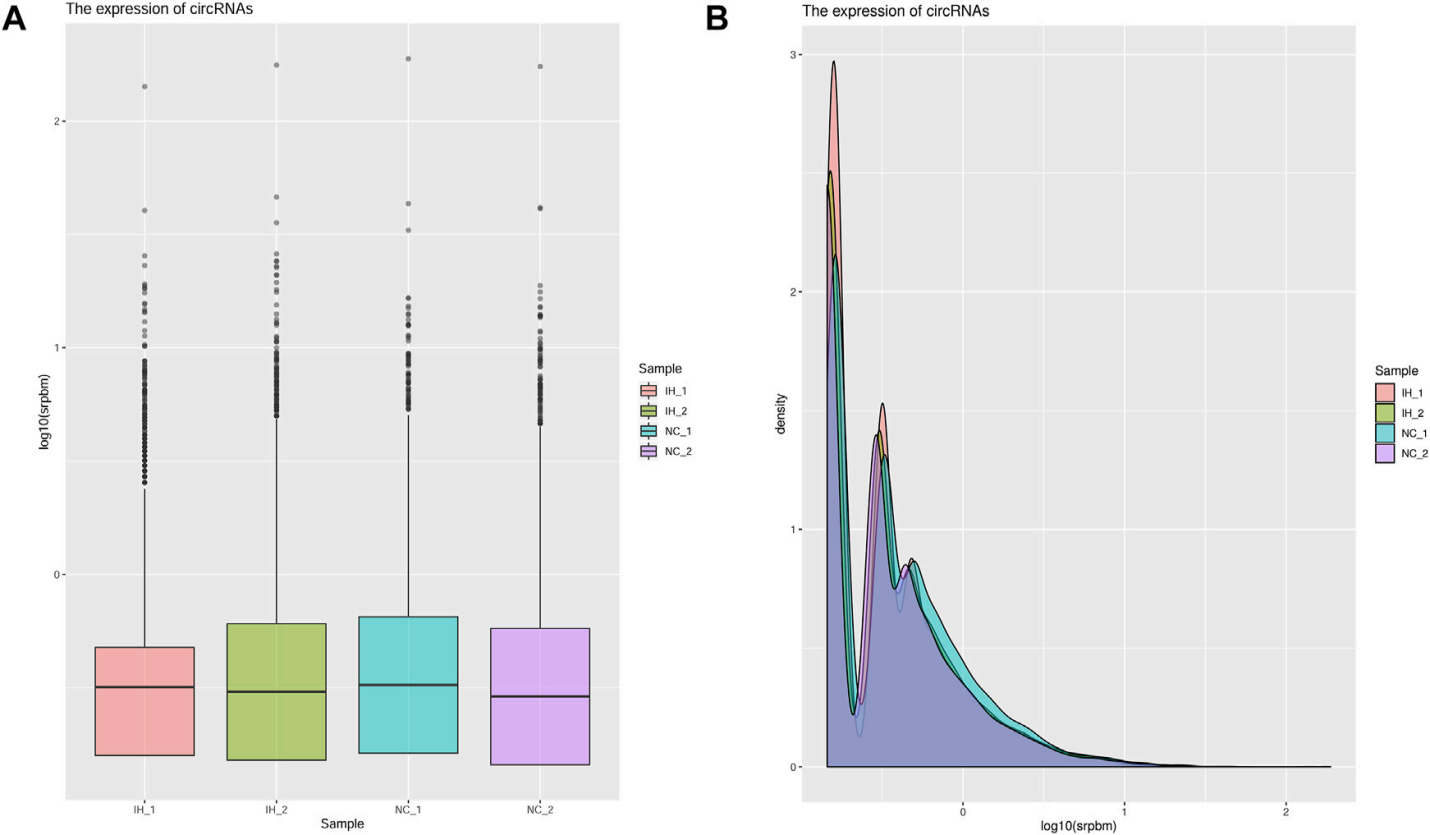
Analysis the expression of circRNAs in each sample. **(A)** The boxplot of the circRNA expression in each sample. **(B)** The density of the circRNA expression in each sample.

The circRNA number and the corresponding circRNA-hosting gene number in each sample were presented in Supplementary Table S2, and a total of 22,362 circRNAs were identified. The box plot showed that the distribution of the circRNAs expression profiles across all the samples was almost identical (Figure 2A). Results of analyses of the distribution density of circRNAs expression in each sample indicated that there were no significant differences between two groups (Figure 2B). Additionally, circRNAs were divided into three types according to the source, including exonic circRNA (circRNA), intronic circRNA (ciRNA), and intergenic. The proportion of circRNA type in each sample was displayed in Supplementary Figure S1. The most common type of candidate circRNA was obviously derived from exonic regions.

### Identification of DE circRNAs

**FIGURE 3:**
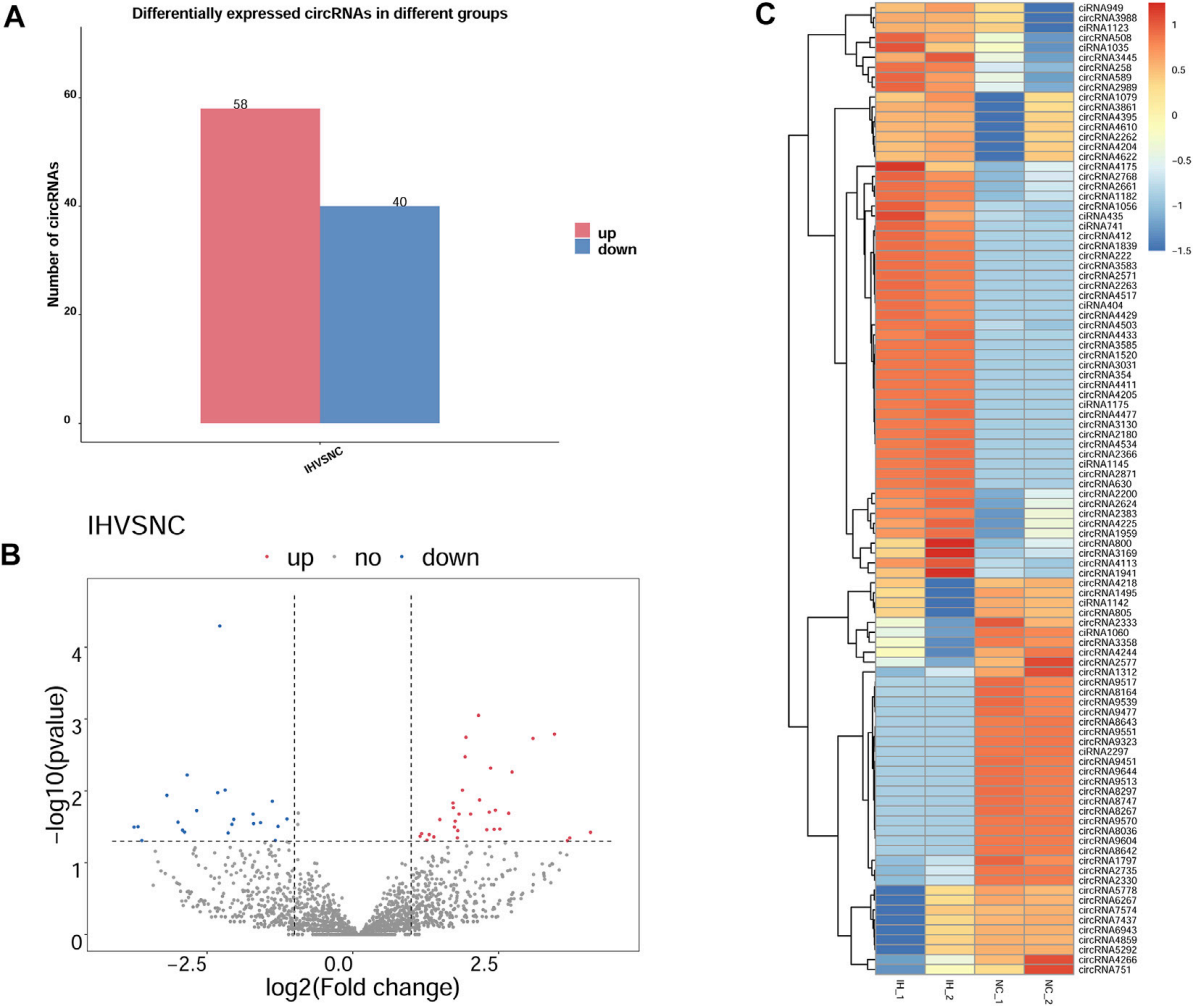
DE circRNAs between IH group and NC group. **(A)** Bar graph for total number of up- and down-regulated DE circRNAs based on |log2 (fold-change)| ≥1 and *p* value < 0.05. **(B)** Volcano plot of DE circRNAs between IH group and NC group, blue dots representing the down-regulated circRNAs, red dots representing the up-regulated circRNAs in IH samples, and the gray dots representing the ones with no differences. **(C)** Heat map generated by hierarchical clustering of DE circRNAs in IH group and NC group, red representing high expressed circRNAs and blue for low expressed circRNAs.

As histogram (Figure 3A) presented, 98 circRNAs were identified as being dysregulated in IH-treated BRL-3A cells with a |log2 (fold-change)| ≥1 and *p* value < 0.05. Among them, 58 were up-regulated and 40 were down-regulated in IH group compared with NC group. Next, a volcano plot was constructed using fold change values and *p* values for visualizing differential expression of circRNAs between the IH and NC groups (Figure 3B). The DE circRNAs were further displayed in the hierarchical clustering (Figure 3C), with red and blue colors indicating high and low expression levels. The results of hierarchical clustering revealed that the expression patterns of circRNA were distinguishable between the NC and IH groups. The differentially expressed up-regulated and down-regulated circRNAs by log2 (fold-change) value are summarized in Supplementary Table S3. Taken together, these data suggested that the expression patterns of circRNA in IH group were different from those in NC group.

### Gene ontology and kyoto encyclopedia of genes and genomes analyses

**FIGURE 4:**
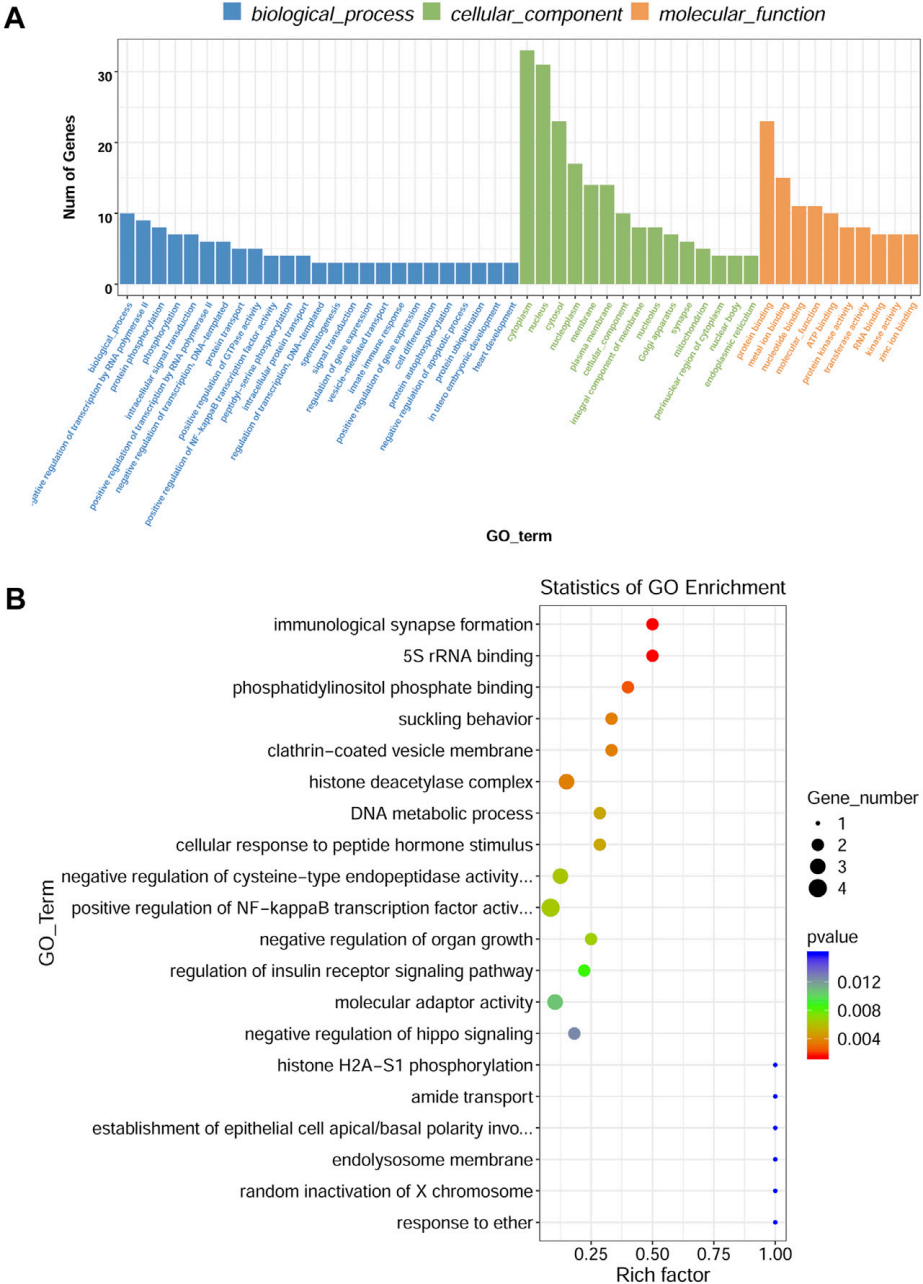
GO enrichment analysis of DE circRNA-hosting gene. **(A)** The top 25 GO terms in the biological process, top 15 GO terms in the cellular component, and top 10 GO terms in the molecular function. **(B)** Scatter plot of GO enrichment analysis, X-axis representing rich factor, Y-axis representing GO_term.

**FIGURE 5:**
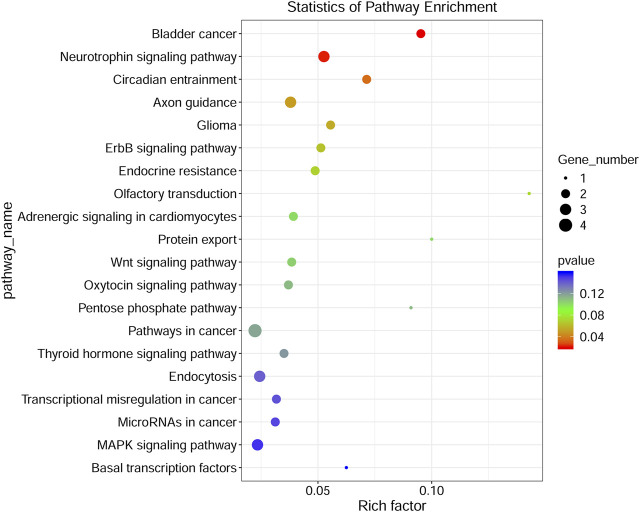
KEGG pathway analyses of DE circRNA-hosting genes. The color of the dot represented the *p* value, the size of the dot represented the number of genes, Y-axis showed pathway_name, X-axis showed rich factor.

To investigate the functions of DE circRNA host genes in IH-induced liver cell injury, GO function enrichment analysis and KEGG pathway enrichment analysis of host genes were performed. The most significant GO terms in the biological process (top 25), cellular component (top 15), and molecular function (top 10) were presented in Figure 4A. The scatter plot of GO enrichment analysis was presented in Figure 4B. The DE circRNA host genes were mainly enriched in biological_process, negative regulation of transcription by RNA polymerase II, protein phosphorylation, phosphorylation, intracellular signal transduction, positive regulation of transcription by RNA polymerase II, negative regulation of transcription, DNA−templated, protein transport, positive regulation of GTPase activity, and positive regulation of NF−kappaB transcription factor activity in biological process; protein binding, metal ion binding, nucleotide binding, molecular_function, ATP binding, protein kinase activity, transferase activity, RNA binding, kinase activity, and zinc ion binding in molecular function. The top 20 KEGG signal pathways based on *p* value and rich factor were listed in scatter plots (Figure 5). The DE circRNA host genes were mainly enriched in pathways such as circadian entrainment, Wnt signaling pathway, MAPK signaling pathway, and protein export, etc.

### qRT-PCR validation of circRNA expression

**FIGURE 6:**
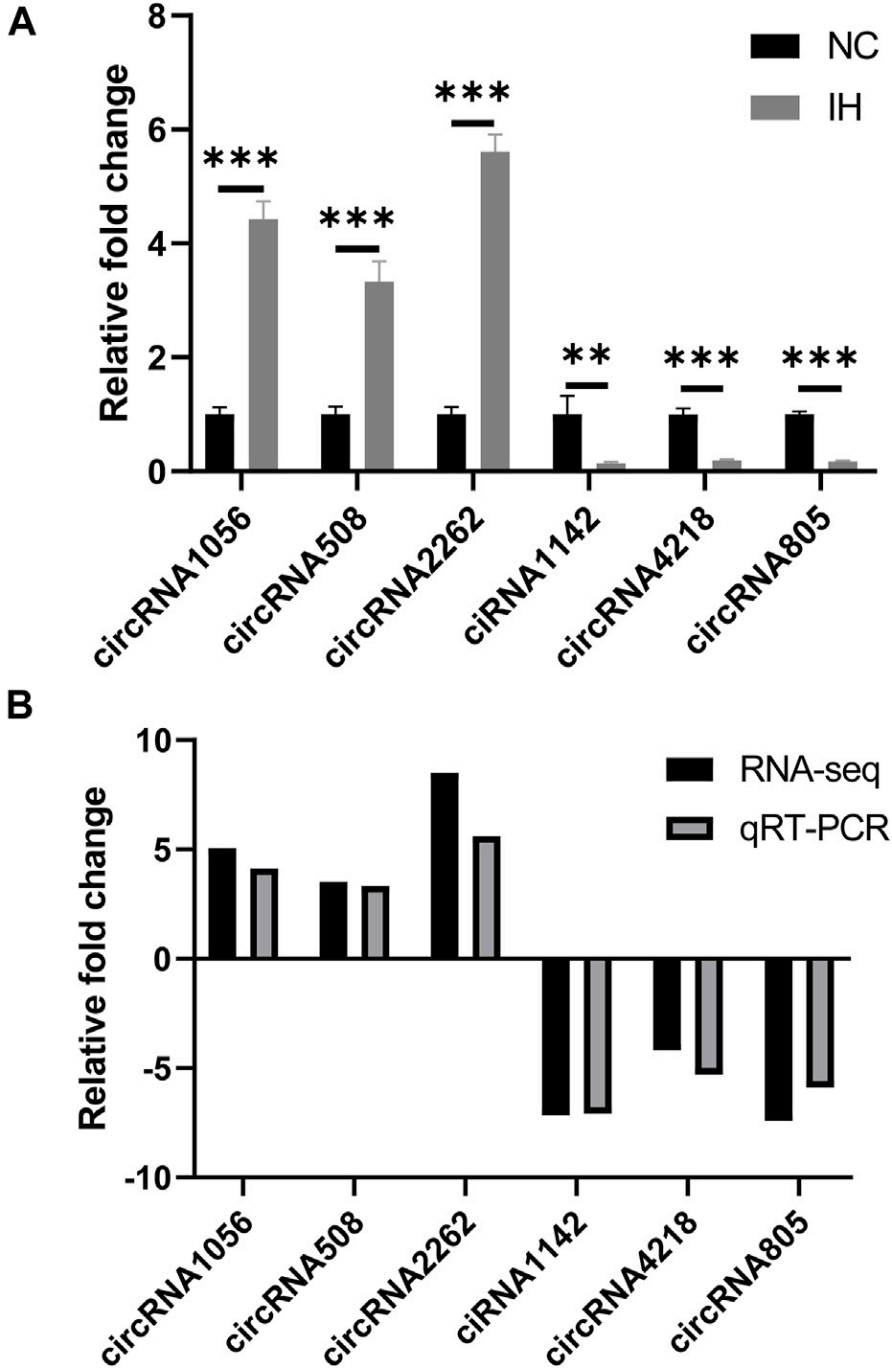
qRT-PCR validation of circRNA expression. **(A)** The relative expression levels of circRNA1056, circRNA508, circRNA2262, ciRNA1142, circRNA4218, and circRNA805 by qRT-PCR analysis. **(B)** The expression pattern of circRNAs in both qRT-PCR and RNA-seq. **p < 0.01; ***p < 0.001.

3 up-regulated circRNAs (circRNA1056, circRNA508, and circRNA2262) and 3 down-regulated circRNAs (ciRNA1142, circRNA4218, and circRNA805) were selected for validation because they had high number of back-splicing junction reads. The results showed that the relative expression of circRNA1056, circRNA508 and circRNA2262 in IH group were significantly higher than those in NC group and ciRNA1142, circRNA4218, and circRNA805 were significantly decreased (Figure 6A). This was agreement with the sequencing data (Figure 6B).

### Construction of a circRNA-miRNA-mRNA regulatory network

**FIGURE 7:**
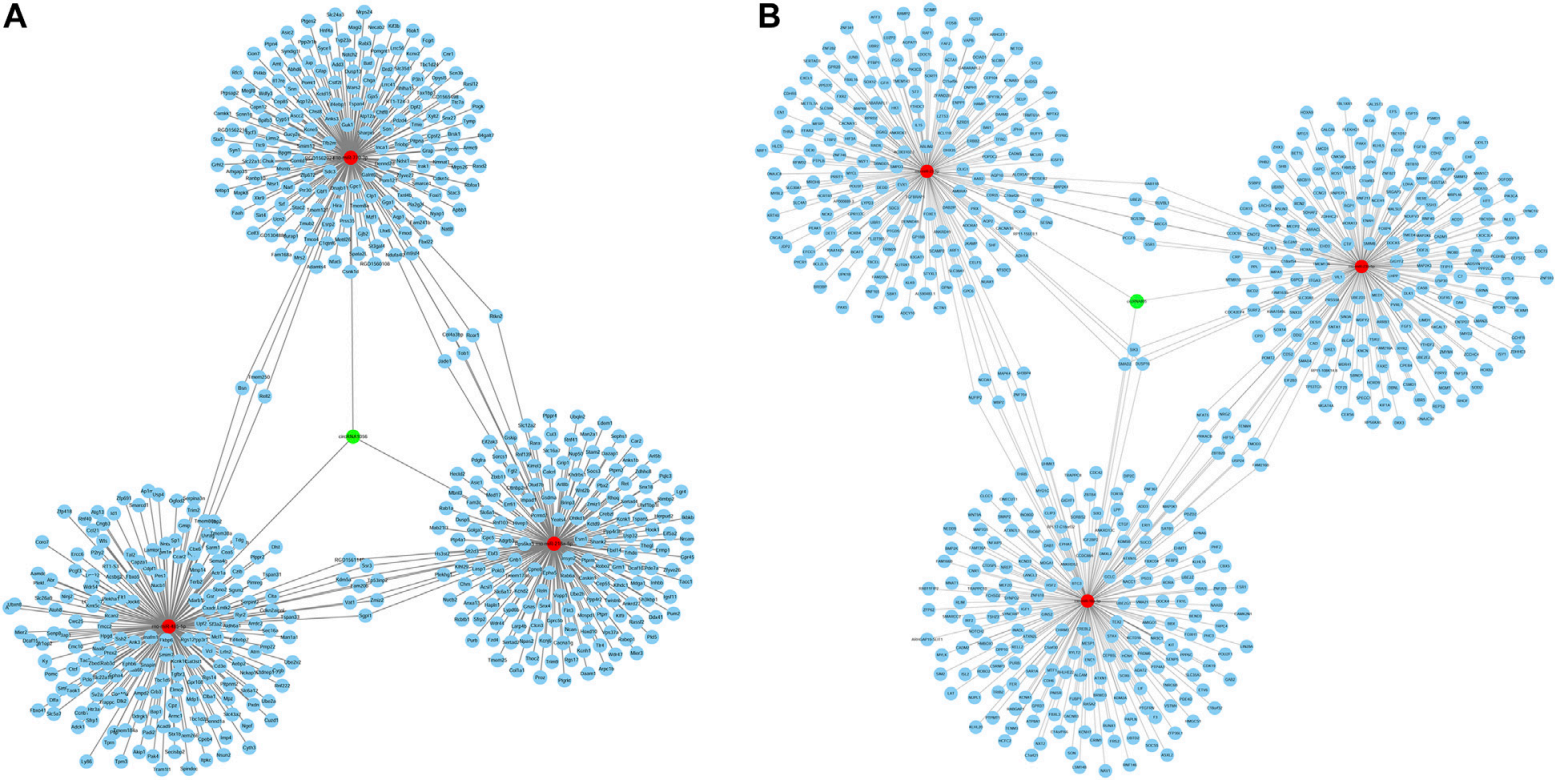
CircRNA-miRNA-mRNA regulatory network of circRNA1056 **(A)** and circRNA805 **(B)**. Green nodes are DE circRNA, red nodes are miRNAs, and blue nodes are target mRNAs.

CircRNAs have been demonstrated to act as miRNA sponge, which can regulate downstream genes expression by competing with miRNAs. Hence, the circRNA–miRNA–mRNA interaction networks were established to show a complex interaction between circRNAs, miRNAs, and mRNA. A total of 26 miRNAs and 22 miRNAs (data not shown) were predicted to be targeted by circRNA1056 and circRNA805, respectively. Their top 3 predicted target miRNAs and corresponding target mRNAs are selected to be displayed in Figure 7.

## Discussion

Our study revealed that 98 circRNAs were identified to be dysregulated in IH-treated BRL-3A cells. Further bioinformatics analysis revealed that the DE circRNAs were involved in various physiologic processes such as positive regulation of NF−kappaB transcription factor activity, protein phosphorylation. The pathway of KEGG enrichment includes various pathway such as Wnt signaling pathway and MAPK signaling pathway. The circRNA-miRNA-mRNA regulatory networks were also constructed based on the circRNA-miRNA and miRNA-mRNA interaction prediction. Collectively, our findings provided an overview for the role circRNAs in the IH-induced liver cell injury and might assist future pathophysiology researches in OSA-related liver injury.

OSA has been demonstrated to be a vital risk factor for liver injury and NAFLD (Musso et al., 2012; Mesarwi et al., 2019). A multi-center cross-sectional study including 1,285 patients with suspected OSA revealed that OSA severity were independently associated with liver cytolysis and liver steatosis (Trzepizur et al., 2016). Our previous meta-analysis enrolling 9 cross-sectional studies with 1,133 pediatric patients found that OSA was associated with NAFLD evidenced by progressive liver fibrosis and elevated liver enzymes (Chen et al., 2020a). A few studies have been performed to investigate the underlying mechanisms (Zhen et al., 2015; Chen et al., 2021). Nevertheless, the potential molecular mechanisms are still far from conclusion. *In vitro* model of IH mimicking OSA has been established and validated by accumulating studies (Berger et al., 2013; Zhen et al., 2015; Singh et al., 2019). Zhen et al. (Zhen et al., 2015) exposed to liver cells to IH and found that IH inhibited liver cell proliferation and accelerated cell apoptosis. Similar to their results, our study also indicated that IH resulted in liver cell injury *in vitro* evidenced by decreased cell viability and cell density, and aggravated cell apoptosis rate.

Previous studies have shown that circRNAs participate in the development of various liver diseases. Wang et al. (Wang et al., 2020d) demonstrated that p66Shc was upregulated in APAP-induced liver injury. Circ-CBFB functioned as the sponge of miR-185-5p to trigger mitochondrial dynamics perturbation via p66Shc. Another study exploring the role of circRNA in radiation-induced liver disease showed that circRSF1 enhanced RAC1 expression by acting as miR-146a-5p sponge to inhibit miR-146a-5p, and thus increased the cell viability, and promoted fibrotic and inflammatory phenotype of irradiated LX2 cells (Chen et al., 2020b). As regarding to NAFLD, Guo et al. (Guo et al., 2018) found that circRNA_0046366 abolished the inhibitory effect of miR-34a on PPAR, and then improved the steatosis-related triglyceride metabolism. Jin and colleagues (Jin et al., 2020)reported that circRNA_002581-miR-122-CPEB1 axis actively participated in the pathogenesis of non-alcoholic steatohepatitis via PTEN-AMPK-mTOR pathway-related autophagy suppression. Although circRNAs play important roles in various liver diseases, their contribution to OSA-related liver injury remains unknown.

In the present study, DE cirRNAs were found to be enriched in GO term “positive regulation of NF−kappaB transcription factor activity in biological process”. This finding was supported by previous animal studies (Savransky et al., 2007; Kang et al., 2017). Savransky et al. (Savransky et al., 2007) exposed mice to CIH or intermittent air for 12 weeks and found that CIH resulted in increase of serum ALT and increased active NF−kappaB in the nuclear fraction of hepatocytes. In addition, the pathway analysis indicated DE cirRNAs were involved in Wnt signaling pathway, MAPK signaling pathway. Kang et al. (Kang et al., 2017) found that CIH led to diet-induced obesity (DIO) mice liver fibrosis. Significantly higher protein expression levels of MyD88, TLR4, phosphorylated (phospho-) I-κB, phospho-ERK1/2 activation, and NF-kB in the liver were observed in CIH group than those in the controls. The results suggested that CIH induced liver fibrosis by TLR4/MyD88/MAPK/NF-kB signaling pathways in DIO mice. An animal study reported that Wnt/β-catenin signaling pathway abnormalities was observed in hippocampus in a mouse model of CIH-induced cognitive deficits and that LiCl might alleviate CIH-induced cognitive damage by Wnt/β-catenin signaling pathway (Pan et al., 2016). We speculate that the mechanisms mediating OSA-related liver injury are complex and multiple signaling pathways are involved in this process. DE circRNAs may exert a regulatory role in controlling multiple signaling pathways.

This study had several strengths. First of all, to our best knowledge, this was the first study investigating the role of circRNAs in OSA-related liver diseases. Secondly, the expression profile of circRNAs was detected by high-throughput sequencing technology in this study. Some studies demonstrated the advantages of sequencing technology versus array technology as it showed substantially enhanced sensitivity in detecting ability (Marioni et al., 2008; Brennan et al., 2012). Thirdly, the findings of our study supplied a fundamental support for further research in regulatory role of circRNAs as ceRNAs in the IH-induced liver injury and had also provided a new direction for the study of OSA-related liver injury. Despite the above advantages, some limitations in our study should be addressed. Firstly, Although IH is an important feature of OSA, it is limited by the absence of other feature such as transthoracic pressure swings, hypercapnia, and sympathetic hyperactivity. Secondly, although the expression profile of DE circRNAs has been determined and their potential role was analyzed, the exact mechanisms of these DE circRNAs in IH-induced liver injury were not explored in this study. Thus, more functional studies are required to confirm the precise molecular regulatory mechanisms of circRNAs. Thirdly, only one liver cell line was used to evaluate IH treatment effect on liver cell. The use of two liver cell lines would contribute to more robust results. Finally, only two pairs of samples were compared in the present study and more samples would result in solid conclusion. However, the sequencing data was validated by qRT-PCR.

In conclusion, the present study characterized a profile of dysregulated circRNAs in IH-induced BRL-3A cell injury. The CircRNA-miRNA-mRNA regulatory networks were constructed, and bioinformatics analysis revealed that DE circRNAs may exert important roles in the pathology of IH-induced liver injury. The findings provide preliminary support for further research in mechanisms and a new theory for the pathogenesis of OSA-related liver injury.

## Data Availability

The data presented in the study are deposited in the NCBI SRA database repository, accession number PRJNA872867.

## Abbreviations

IH, intermittent hypoxia; OSA, obstructive sleep apnea; qRT-PCR, quantitative real-time PCR; circRNAs, circular RNAs; CCK-8, cell counting kit-8; KEGG, Kyoto Encyclopedia of Genes and Genomes; GO, gene ontology; CIH, chronic intermittent hypoxia; NAFLD, nonalcoholic fatty liver disease; DE circRNAs, differentially expressed circRNAs; NC, normoxic control; CC, cellular components; BP, biological processes; MF, molecular functions; SD, standard deviation.
