# Supplementary material for: Expression profiling and functional analysis of circular RNAs in vitro model of intermittent hypoxia-induced liver injury
Source: Front Physiol. 2022 Sep 14;13:972407. doi: 10.3389/fphys.2022.972407 (PMC9515621; doi:10.3389/fphys.2022.972407)
Supplement: Supplementary file 1 [file Table1.DOCX]

Table S1 Primers used for qRT-PCR.

| Genes | forward primer | reverse primer |
| --- | --- | --- |
| GAPDH | 5′- CAGTGCCAGCCTCGTCTCAT-3′ | 5′-aggggccatccacagtcttc-3′ |
| circRNA1056 | 5′-GAAGGTGAGGAGAGCACTGG-3′ | 5′-GCTGTAGTCGTCCCTGGTTC-3′ |
| circRNA508 | 5′-TATTGCCACCACTCTGGTCA-3′ | 5′-AGTCCTCCTTTCGCAAGTCA-3′ |
| circRNA2262 | 5′-GTTCCACAGAGGATGGCTGT-3′ | 5′-CCGGTTCTTGATGACTGGAT-3′ |
| ciRNA1142 | 5′-GGTACAGCTTCCGACACCTC-3′ | 5′- GACGCTGTCCCAAGAACAAT-3′ |
| circRNA4218 | 5′-GTCTCTTCGTAGCCGTGGTC-3′ | 5′-CTTCACAGTCAACGCCAACA-3′ |
| circRNA805 | 5′-AAGTGAAGGAAGGAGGGCC-3′ | 5′-ATGGTGTCCATCGGCTCC-3′ |
