# Supplementary figures and images for: Expression profiling and functional analysis of circular RNAs in vitro model of intermittent hypoxia-induced liver injury
Source: Front Physiol. 2022 Sep 14;13:972407. doi: 10.3389/fphys.2022.972407 (PMC9515621; doi:10.3389/fphys.2022.972407)

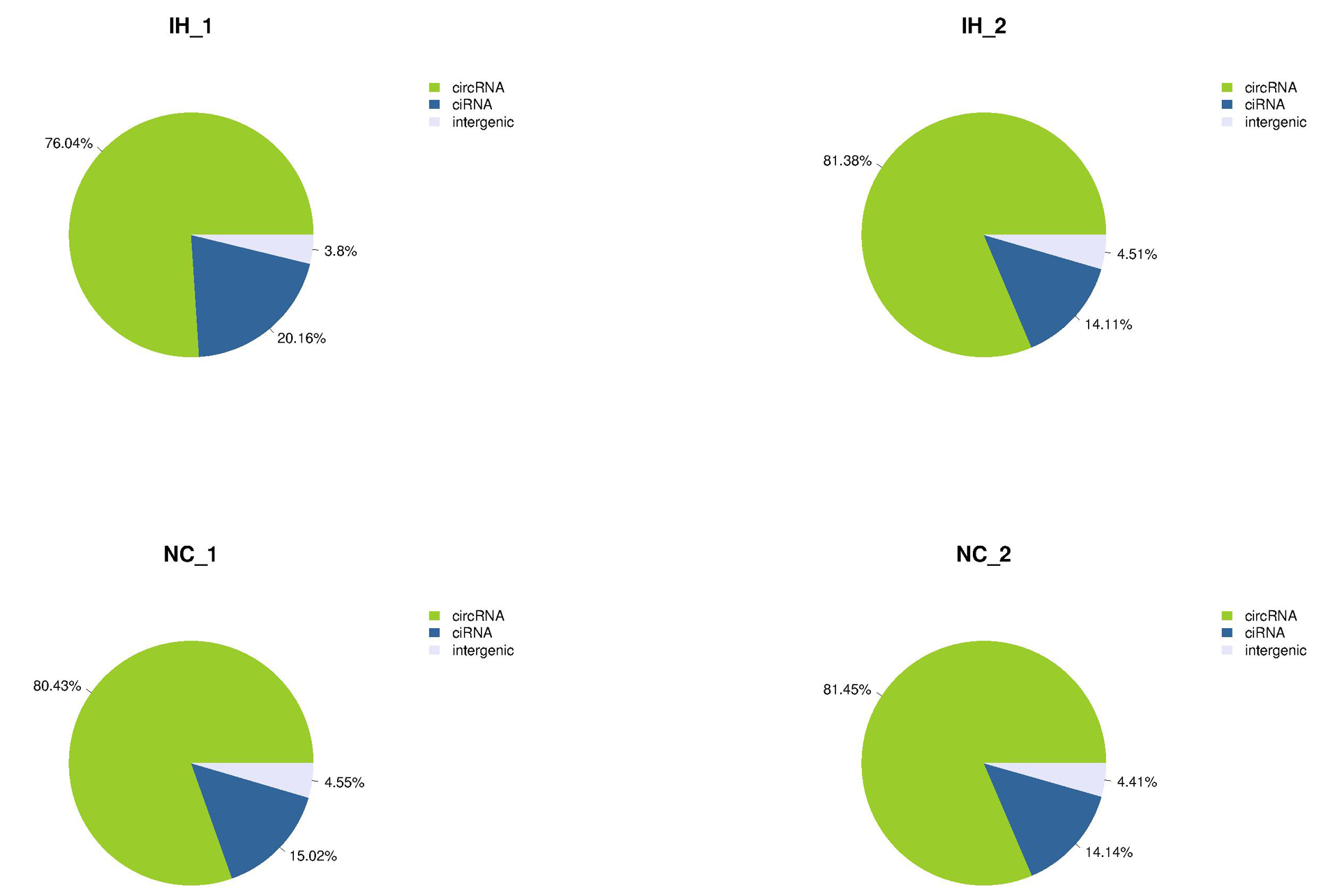

Supplement: Supplementary file 2 [file Image1.TIF]
