# Supplementary material for: Expression profiling and functional analysis of circular RNAs in vitro model of intermittent hypoxia-induced liver injury
Source: Front Physiol. 2022 Sep 14;13:972407. doi: 10.3389/fphys.2022.972407 (PMC9515621; doi:10.3389/fphys.2022.972407)
Supplement: Supplementary file 3 [file Table2.DOCX]

Table S2 Number of circRNAs and circRNA-hosting genes in each sample

| Sample | IH_1 | IH_2 | NC_1 | NC_2 | Total number |
| --- | --- | --- | --- | --- | --- |
| Candidate back-spliced junction reads | 1200368(2.48%) | 712294(1.41%) | 724088(1.52%) | 1041180(1.95%) | / |
| Confident post reads | 23432(0.05%) | 27964(0.06%) | 21117(0.04%) | 25020(0.05%) | / |
| CircRNA number | 5872 | 6261 | 4573 | 5656 | 22362 |
| CircRNA-hosting gene number | 3052 | 3106 | 2594 | 2988 | 11740 |

Abbreviation: IH=intermittent hypoxia, NC=normoxic control.
