# Supplementary material for: Expression profiling and functional analysis of circular RNAs in vitro model of intermittent hypoxia-induced liver injury
Source: Front Physiol. 2022 Sep 14;13:972407. doi: 10.3389/fphys.2022.972407 (PMC9515621; doi:10.3389/fphys.2022.972407)
Supplement: Supplementary file 4 [file Table3.DOCX]

Table S3: List of dys-regulated circRNAs in IH group as compared to NC group

| CircRNA | log2 (fold-change) | Regulation | p-value | q-value | Chromosome | circRNA_type | Gene Name |
| --- | --- | --- | --- | --- | --- | --- | --- |
| circRNA4411 | inf | up | 0.00 | 0.52 | chr8 | circRNA | Map2k5 |
| circRNA2871 | inf | up | 0.00 | 0.52 | chr2 | circRNA | Sclt1 |
| ciRNA404 | inf | up | 0.00 | 0.74 | chr7 | ciRNA | Akap8 |
| circRNA630 | inf | up | 0.00 | 0.82 | chr10 | circRNA | Gpatch8 |
| ciRNA1145 | inf | up | 0.00 | 0.82 | chr8 | ciRNA | Sik2 |
| circRNA2366 | inf | up | 0.01 | 1 | chr4 | circRNA | Chn2 |
| circRNA4429 | inf | up | 0.01 | 1 | chr12 | circRNA | Gtf2i |
| circRNA4534 | inf | up | 0.01 | 1 | chr12 | circRNA | N4bp2l2 |
| circRNA3130 | inf | up | 0.02 | 1 | chr1 | circRNA | Prr12 |
| ciRNA741 | inf | up | 0.02 | 1 | chr2 | ciRNA | Lmna |
| circRNA4517 | inf | up | 0.02 | 1 | chr10 | circRNA | Ncor1 |
| circRNA3585 | inf | up | 0.03 | 1 | chr9 | circRNA | Gulp1 |
| ciRNA1175 | inf | up | 0.03 | 1 | chr6 | ciRNA | Ltbp2 |
| circRNA4477 | inf | up | 0.03 | 1 | chr3 | circRNA | Dennd1a |
| circRNA412 | inf | up | 0.03 | 1 | chr10 | circRNA | Clec16a |
| circRNA4433 | inf | up | 0.04 | 1 | chr12 | circRNA | Baiap2l1 |
| circRNA4205 | inf | up | 0.04 | 1 | chr13 | intergenic | intergenic_circRNA |
| circRNA354 | inf | up | 0.04 | 1 | chr11 | circRNA | Acap2 |
| circRNA3031 | inf | up | 0.04 | 1 | chr1 | circRNA | Nhsl1 |
| circRNA1520 | inf | up | 0.04 | 1 | X | circRNA | Wwc3 |
| circRNA3583 | inf | up | 0.04 | 1 | chr9 | circRNA | Nck2 |
| circRNA2571 | inf | up | 0.04 | 1 | chr3 | circRNA | Ubr3 |
| circRNA222 | inf | up | 0.04 | 1 | chr12 | circRNA | Mapkapk5 |
| circRNA1839 | inf | up | 0.04 | 1 | chr6 | circRNA | Dtnb |
| circRNA2180 | inf | up | 0.04 | 1 | chr5 | circRNA | Zmym4 |
| circRNA2263 | inf | up | 0.05 | 1 | chr5 | circRNA | Rere |
| circRNA1079 | 4.08 | up | 0.04 | 1 | chr14 | circRNA | LOC680039 |
| ciRNA949 | 3.72 | up | 0.05 | 1 | chr9 | ciRNA | LOC100359583 |
| circRNA3861 | 3.68 | up | 0.05 | 1 | chr8 | circRNA | Ube3d |
| circRNA3988 | 3.46 | up | 0.00 | 0.82 | chr15 | circRNA | Ube2e2 |
| circRNA2262 | 3.09 | up | 0.00 | 0.82 | chr5 | circRNA | Rere |
| circRNA4395 | 2.73 | up | 0.01 | 1 | chr7 | circRNA | Tatdn1 |
| circRNA4204 | 2.67 | up | 0.02 | 1 | chr12 | intergenic | intergenic_circRNA |
| ciRNA1123 | 2.52 | up | 0.03 | 1 | chr15 | ciRNA | Fbxo34 |
| circRNA1959 | 2.45 | up | 0.02 | 1 | chr6 | circRNA | Rps6ka5 |
| circRNA4610 | 2.42 | up | 0.03 | 1 | chr15 | circRNA | R3hcc1 |
| circRNA2200 | 2.36 | up | 0.00 | 1 | chr5 | circRNA | Pum1 |
| circRNA1056 | 2.34 | up | 0.02 | 1 | chr14 | circRNA | Scfd2 |
| circRNA4622 | 2.30 | up | 0.03 | 1 | chr7 | circRNA | Mdm2 |
| circRNA1941 | 2.17 | up | 0.01 | 1 | chr6 | circRNA | Ltbp2 |
| circRNA4225 | 2.16 | up | 0.00 | 0.78 | chr15 | intergenic | intergenic_circRNA |
| ciRNA435 | 2.02 | up | 0.02 | 1 | chr7 | ciRNA | Rpl3 |
| circRNA4113 | 1.94 | up | 0.00 | 0.82 | chr10 | circRNA | Septin9 |
| circRNA2383 | 1.93 | up | 0.00 | 1 | chr4 | circRNA | Prdm5 |
| circRNA2661 | 1.88 | up | 0.01 | 1 | chr3 | circRNA | Fbn1 |
| circRNA508 | 1.82 | up | 0.02 | 1 | chr10 | circRNA | Ankfy1 |
| circRNA4175 | 1.80 | up | 0.04 | 1 | chr1 | intergenic | intergenic_circRNA |
| circRNA3169 | 1.79 | up | 0.05 | 1 | chr1 | circRNA | Lrrc28 |
| ciRNA1035 | 1.76 | up | 0.03 | 1 | chr6 | ciRNA | Babam2 |
| circRNA3445 | 1.74 | up | 0.03 | 1 | chr1 | circRNA | Dock8 |
| circRNA589 | 1.72 | up | 0.02 | 1 | chr10 | circRNA | Scpep1 |
| circRNA258 | 1.72 | up | 0.01 | 1 | chr12 | circRNA | Ttc28 |
| circRNA4503 | 1.49 | up | 0.03 | 1 | chr5 | circRNA | Ror1 |
| circRNA1182 | 1.39 | up | 0.04 | 1 | chr19 | circRNA | Slc10a7 |
| circRNA2624 | 1.31 | up | 0.04 | 1 | chr3 | circRNA | Ldlrad3 |
| circRNA2989 | 1.27 | up | 0.05 | 1 | chr2 | circRNA | Camk2d |
| circRNA800 | 1.18 | up | 0.04 | 1 | chr17 | circRNA | Etl4 |
| circRNA2768 | 1.15 | up | 0.04 | 1 | chr2 | circRNA | Ap3b1 |
| circRNA9517 | -inf | down | 0.00 | 0.85 | chr2 | intergenic | intergenic_circRNA |
| circRNA8747 | -inf | down | 0.00 | 1 | chr5 | circRNA | Eri3 |
| circRNA9539 | -inf | down | 0.00 | 1 | chr8 | intergenic | intergenic_circRNA |
| circRNA9323 | -inf | down | 0.01 | 1 | chr9 | circRNA | Hdlbp |
| ciRNA2297 | -inf | down | 0.01 | 1 | chr13 | ciRNA | Disp1 |
| circRNA8267 | -inf | down | 0.01 | 1 | chr16 | circRNA | Mtus1 |
| circRNA8643 | -inf | down | 0.01 | 1 | chr6 | circRNA | Babam2 |
| circRNA9451 | -inf | down | 0.01 | 1 | chr7 | circRNA | Fkbp11 |
| circRNA9604 | -inf | down | 0.02 | 1 | chr2 | circRNA | Arhgef11 |
| circRNA8642 | -inf | down | 0.02 | 1 | chr6 | circRNA | Memo1 |
| circRNA8036 | -inf | down | 0.02 | 1 | chr12 | circRNA | Gtf2i |
| circRNA9477 | -inf | down | 0.03 | 1 | chr4 | circRNA | Srpk2 |
| circRNA8164 | -inf | down | 0.03 | 1 | chr10 | circRNA | Suz12 |
| circRNA9570 | -inf | down | 0.03 | 1 | chr15 | circRNA | Rpph1 |
| circRNA9551 | -inf | down | 0.03 | 1 | chr16 | circRNA | Wwc2 |
| circRNA8297 | -inf | down | 0.04 | 1 | chr15 | circRNA | Pspc1 |
| circRNA9513 | -inf | down | 0.04 | 1 | chr2 | intergenic | intergenic_circRNA |
| circRNA9644 | -inf | down | 0.04 | 1 | chr1 | circRNA | Plekha7 |
| circRNA1797 | -1.13 | down | 0.02 | 1 | chr6 | circRNA | Ttc7a |
| circRNA751 | -1.28 | down | 0.03 | 1 | chr17 | circRNA | Cdyl |
| circRNA2577 | -1.33 | down | 0.05 | 1 | chr3 | circRNA | Tlk1 |
| circRNA2333 | -1.38 | down | 0.01 | 1 | chr4 | circRNA | AABR07060133 |
| circRNA4244 | -1.58 | down | 0.03 | 1 | chr18 | intergenic | intergenic_circRNA |
| circRNA2330 | -1.70 | down | 0.03 | 1 | chr4 | circRNA | Nrf1 |
| circRNA4266 | -1.71 | down | 0.02 | 1 | chr2 | intergenic | intergenic_circRNA |
| circRNA1312 | -2.04 | down | 0.02 | 1 | chr18 | circRNA | Hars2 |
| circRNA4218 | -2.07 | down | 0.03 | 1 | chr14 | intergenic | intergenic_circRNA |
| circRNA3358 | -2.14 | down | 0.04 | 1 | chr1 | circRNA | Zranb1 |
| circRNA2735 | -2.19 | down | 0.01 | 1 | chr3 | circRNA | Nelfcd |
| ciRNA1060 | -2.28 | down | 0.00 | 0.22 | chr8 | ciRNA | Sema3f |
| circRNA7574 | -2.32 | down | 0.01 | 1 | chr3 | circRNA | Phf21a |
| circRNA4859 | -2.68 | down | 0.02 | 1 | chr12 | circRNA | Pitpnb |
| ciRNA1142 | -2.84 | down | 0.01 | 1 | chr14 | ciRNA | Fryl |
| circRNA805 | -2.89 | down | 0.04 | 1 | chr17 | circRNA | Arhgap21 |
| circRNA5292 | -2.92 | down | 0.04 | 1 | chr16 | circRNA | Fat1 |
| circRNA7437 | -3.00 | down | 0.03 | 1 | chr8 | circRNA | Sema3f |
| circRNA6943 | -3.19 | down | 0.01 | 1 | chr1 | circRNA | Il16 |
| circRNA6267 | -3.62 | down | 0.05 | 1 | chr5 | circRNA | H6pd |
| circRNA1495 | -3.68 | down | 0.03 | 1 | chr20 | circRNA | Sec63 |
| circRNA5778 | -3.75 | down | 0.03 | 1 | X | circRNA | Mid2 |

Abbreviation: NC=normoxic control, IH=intermittent hypoxia.
